# A novel method of manual positioning based on anatomical mark (shoulder-to-shoulder) to prevent postoperative leg-length discrepancy for femoral neck fractures in hip arthroplasty

**DOI:** 10.3389/fsurg.2022.1030657

**Published:** 2022-11-01

**Authors:** Jin-shan Zhang, Yong-qiang Zheng, Xiao-feng Liu, Yong-quan Xu, Yang-zhen Fang, Zhen-yu Lin, Liang Lin, You-jia Xu

**Affiliations:** ^1^ Department of Orthopedics, The Second Affiliated Hospital of Soochow University, Jiangsu, China; ^2^ Department of Orthopedics, Jinjiang Municipal Hospital, Fujian, China

**Keywords:** manual positioning, anatomical mark, “shoulder-to-shoulder”, femoral neck fracture, hip arthroplasty, leg length discrepancy

## Abstract

**Objective:**

To determine whether the two lower extremities are of equal length after hip arthroplasty for femoral neck fractures, we developed a novel method of manual positioning based on anatomical mark (shoulder-to-shoulder) in hip arthroplasty.

**Methods:**

Patients with femoral neck fractures requiring hip arthroplasty from July 2020 to March 2022 in the orthopedic department of Jinjiang Municipal Hospital, Fujian Province, China were recruited. Hip arthroplasty was performed using the proposed “shoulder-to-shoulder” method of manual positioning based on anatomical mark in 52 patients with femoral neck fractures who met the inclusion criteria. “Shoulder-to-shoulder” was achieved by alignment of the marked femoral “shoulder” and the “shoulder” of prosthesis stem. There were 16 male and 36 female patients, with 27 undergoing total hip arthroplasty (THA) and 25 undergoing hip hemiarthroplasty (HA). The fractures were categorized according to the Garden classification: type II, type III, and type IV in 5, 11, and 36 patients, respectively. The vertical distance from the apex of the medial margin of the femoral trochanter to the tear drop line on both sides which was regarded as the length of both limbs were compared *via* postoperative imaging, and the apex–shoulder distance on the ipsilateral side measured *via* postoperative imaging was compared with those measured intraoperatively.

**Results:**

All patients completed the surgery successfully. The measurement results for the lower extremities after THA were as follows: contralateral group, 43.87 ± 5.59 mm; ipsilateral group, 44.64 ± 5.43 mm. The measurement results for the lower extremities after HA were as follows: contralateral group, 45.18 ± 7.82 mm; ipsilateral group, 45.16 ± 6.43 mm. The measurement results for the lower extremities after all arthroplasties were as follows: contralateral group, 44.50 ± 6.72 mm; ipsilateral group, 44.89 ± 5.90 mm. The results for the apex–shoulder distance were as follows: postoperative imaging, 19.44 ± 3.54 mm; intraoperative apex–shoulder distance, 27.28 ± 2.84 mm. Statistical analysis results indicated no statistically significant difference in the postoperative bilateral lower extremity length after hip arthroplasty (*P* = 0.75), while a statistically significant difference was found between the intraoperative and postoperative imaging measurements of the apex–shoulder distance (*P* < 0.01).

**Conclusion:**

The novel method of manual positioning based on anatomical mark (shoulder-to-shoulder) for femoral neck fractures in hip arthroplasty is simple and accurate, making it effective for preventing postoperative bilateral leg length discrepancy.

## Introduction

Hip arthroplasty for displaced femoral neck fractures has achieved remarkable results in relieving pain and rapidly restoring hip function and has become the main treatment modality in elderly population (1, 2). However, there exists the defect of leg length discrepancy (LLD) after hip arthroplasty, it can lead to serious complications, such as limping, back and leg pain, prosthesis loosening, and dislocation (3–7). In addition, the expectations of the patients might usually be high and LLD could be associated with patient dissatisfaction or even legal problems (8, 9). Nevertheless, it could be a challenge for the surgeon to restore the length equality of the two lower limbs, as the destruction caused by the fracture makes it difficult to apply an appropriate anatomical reference during the surgery (10).

Hence, we recruited patients who had femoral neck fractures requiring hip arthroplasty in the study, and the proposed “shoulder-to-shoulder” method of manual positioning based on anatomical mark was used during surgery, which was simple and fast. “Shoulder-to-shoulder” was achieved by alignment of the marked femoral “shoulder” and the “shoulder” of prosthesis stem. The length of the two lower limbs was compared *via* postoperative imaging, and the apex–shoulder distance on the ipsilateral side measured *via* postoperative imaging was compared with those measured intraoperatively.

A new method of manual positioning based on anatomical mark (shoulder-to-shoulder) was proposed for reducing the bilateral leg length discrepancy after hip arthroplasty for femoral neck fractures.

## Methods

As the study subjects, 52 patients with femoral neck fractures requiring hip arthroplasty and meeting the inclusion criteria who were treated in the orthopedic department of Jinjiang Municipal Hospital, Fujian Province, China from July 2020 to February 2022 were recruited. The study was approved by the Institutional Ethics Committee and was performed in accordance with ethical standards (No: jjsyyyxll-2020022). Inclusion criteria of THA were patients without significant functional limitation or cognitive impairment before the fracture, and inclusion criteria of HA were poor mobility; cognitive impairment; patients above 80 years. Exclusion criteria were fractures with hip dysplasia; osteoarthritis; aseptic necrosis of the femoral head; trauma or surgery resulting in deformity affecting accurate measurement; comorbidities of hemiplegia or neurogenic diseases; and HA for contralateral hip. There were 16 male and 36 female patients, with 27 undergoing THA and 25 undergoing HA. The mean age was 78.88 ± 10.02 (58–97) years. The fractures were categorized according to the Garden classification: type II, type III, and type IV in 5, 11, and 36 patients, respectively (Table 1).

**Table 1:** Demographics and baseline characteristics of the patients.

Variable	THA	HA	Total
Sex, no.			
Male	7	9	16
Female	20	16	36
Garden fracture classification, no.			
II	4	1	5
III	4	7	11
IV	19	17	36
Age, years (mean ± SD)	72.89 ± 8.14	85.36 ± 7.60	78.88 ± 10.03

**Figure 1:**
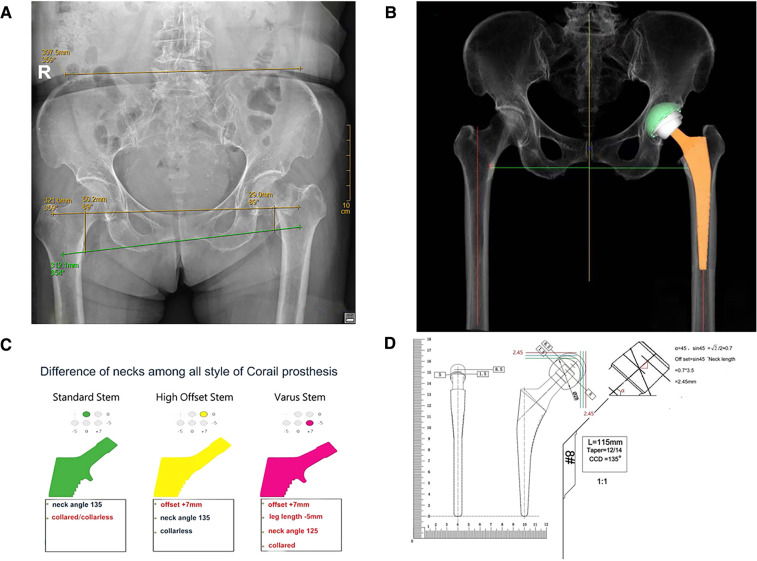
(**A**) Preoperative orthoptic pelvic x-ray measurement: the line connecting the lower edge of the Köhler teardrops on both sides was used as the pelvic reference line (yellow line), and the vertical distance from the apex of the inner edge of the lesser trochanter to the line connecting the Köhler teardrops on both sides represented the lengths of the lower limbs on both sides (red line); (**B**) the AIHIP software; (**C**) final state after planning by the AIHIP system. (**D**) The actual deviation among same size ball head (short, standard and long) horizontal and vertical.

A preoperative assessment of the affected limbs was performed, and standard anteroposterior x-rays of the pelvis were obtained. The x-ray imaging conditions were as follows: ① the patient was placed in a supine position with both lower limbs straightened and both feet facing inward; ② the “scan area” included the hip joint, proximal femur, pubic bone, sciatic bone, and iliac bone; ③ there was no projection deformity of the femoral neck; and ④ the bone texture of the hip joint was clear and sharp, and the ischial spine was clearly visible. Measure the vertical distance from the apex of the medial edge of the lesser trochanter to the line connecting the Köhler teardrops on both sides. The measured distance represented the lengths of lower limbs on both sides (Figure 1A). An AIHIP system (Beijing Changmugu Medical Technology Co., Ltd.) was used for preoperative planning (Figure 1B). We made selection of the prosthesis style, e.g., standard stem, high offset stem, or varus stem (Figure 1C) before the operation; ball head prosthesis (short, standard and long) in the horizontal and vertical deviation is shown with green, blue, red interval (Figure 1D).

All procedures were performed by the same surgeon using a posterolateral approach in a lateral recumbent position. All prostheses were provided by Johnson & Johnson (USA), including two kinds of femoral stems (Corail, DePuy, Warsaw, IN, USA; Summit, DePuy, Warsaw, IN, USA).

**Figure 2:**
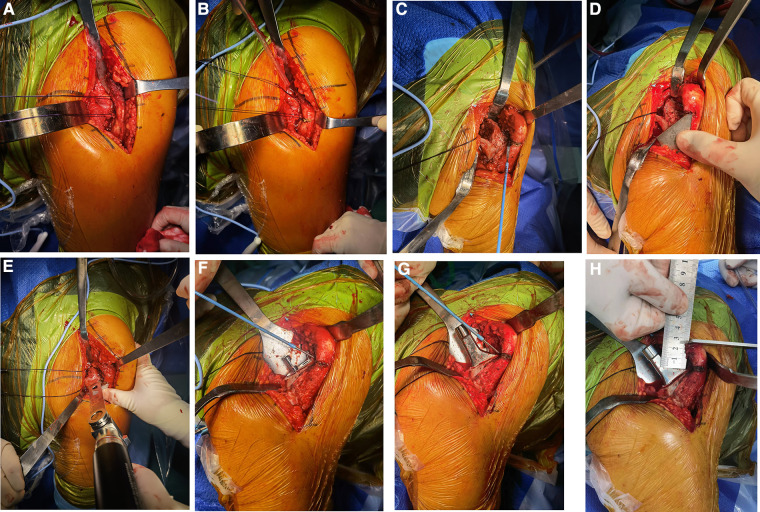
(**A**) The piriformis muscle (blue arrow) and obturator externus muscle (white arrow) were exposed. (**B**) A mark was made using an electric knife under the attachment of obturator externus muscle tendon, where the “shoulder” of femur was marked. (**C**) The mark would be found at the level of the lowest point of the piriformis fossa of the femoral trochanter after the attachment was separated subperiosteally. (**D,E**) Osteotomy would be performed after the apex of the osteotomy template was placed at the “shoulder” of femur. (**F**) The “shoulder” of the medullary cavity file reached the “shoulder” level of the marked femur. (**G**) “Shoulder-to-shoulder” (alignment of the “shoulder” of femur and the “shoulder” of prosthesis stem) was confirmed again after fitting the prosthesis. (**H**) Intraoperative measurement of the apex–shoulder distance.

During surgery, the piriformis muscle and obturator externus muscle were exposed (Figure 2A), and we made a mark using an electric knife under the attachment of obturator externus muscle tendon (Figure 2B). Then, the mark would be found at the level of the lowest point of the piriformis fossa of the femoral trochanter after the attachment was separated subperiosteally, where the “shoulder” of femur was marked (Figure 2C). The type of osteotomy template matching with appropriate femoral stem was selected according to preoperative planning, and osteotomy would be performed after the apex of the osteotomy template was placed at the “shoulder” of femur (Figures 2D,E). During the operation, the rotation center of acetabulum should try to be kept in the position. Based on the maximum diameter of the femoral head taken out, the final file should not exceed the maximum diameter of the femoral head of 6 mm as the upper limit, and the size of the acetabular cup planned by AIHIP as the reference, and did not blindly pursue the use of big head and choose a larger mortar cup, this would lead to too much bone loss and acetabular wall thinning, acetabular wall was often thin biological cup holding firm, not easy to grow into the bone, also easy to cause acetabular cup into the acetabular wall fracture occurred. The medullary cavity files of the femoral side were filed from small to large one by one until the “shoulder” of the medullary cavity file reached the “shoulder” level of the marked femur and it did not sink in the medullary cavity with hammering (Figure 2F); we rotated the medullary cavity file to see whether a good inlay without micromovement, if the stability was satisfactory, the optimal size of the prosthesis was selected according to the trial medullary cavity file, “shoulder-to-shoulder” (alignment of the “shoulder” of femur and the “shoulder” of prosthesis stem) was confirmed again after fitting the prosthesis (Figure 2G). The size and model of AIHIP hip stems, such as standard stem, high offset stem and varus stem (Figure 1C), should be selected appropriately in order to avoid too much reduction or increment of eccentricity, which would affect the stability and the middle gluteal muscle weakness, otherwise lead to excessive wear of acetabular liners, respectively. Finally, a short-end die was used to test the mold (convenient for reduction and dislocation), and a relatively suitable ball head was selected as reference to AIHIP preoperative planning of the ball head.

**Figure 3:**
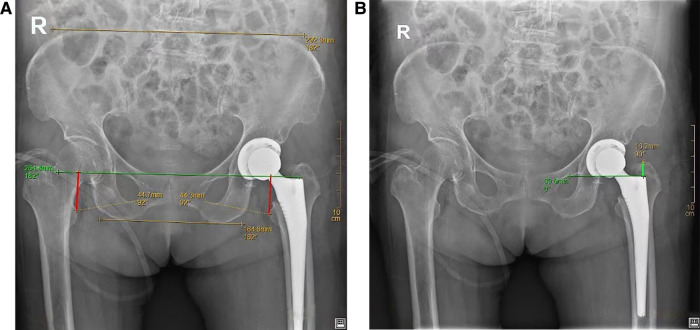
(**A**) Postoperative x-ray measurement of the bilateral leg length discrepancy using the preoperative method (red line); (**B**) postoperative imaging of the ipsilateral apex–shoulder distance (green line).

The lengths of the two lower limbs were compared *via* postoperative imaging measurements, which were performed by measuring the vertical distance from the apex of the medial edge of the lesser trochanter to the line connecting the Köhler teardrops on both sides (indicated by the red line in Figure 3A). The apex–shoulder distance on the ipsilateral side measured *via* postoperative imaging (indicated by the green line in Figure 3B) was compared with those measured during surgery (as shown in Figure 2H). Considering the possible factor of acetabular side, we measured the vertical distance from the center of the contralateral and ipsilateral femoral heads to the line connecting the lower edge of the Köhler teardrop after surgery (indicated by the blue line in Supplementary Figure S1) in the THA group.

SPSS26.0 statistical software was used for analysis. Quantitative data were expressed as mean ± standard deviation, and a *t* test was performed for comparison between groups. The significance level was set at *P* < 0.05.

## Results

The results of bilateral lower limb length measurement after THA were 43.87 ± 5.59 mm for the contralateral group and 44.64 ± 5.43 mm for the ipsilateral group, and the results of bilateral lower limb length measurement after HA were 45.18 ± 7.82 mm for the contralateral group and 45.16 ± 6.43 mm for the ipsilateral group. The results of bilateral lower limb length measurement after all arthroplasties were 44.50 ± 6.72 mm for the contralateral group and 44.89 ± 5.88 mm for the ipsilateral group. A statistical analysis indicated that for both THA and HA, the postoperative bilateral lower limb length difference was not statistically significant (*P* = 0.75) (Table 2). The measurement results for the postoperative imaging apex–shoulder distance on the ipsilateral side were 19.44 ± 3.54 mm, those for the intraoperative apex–shoulder distance were 27.28 ± 2.85 mm. However, there was a statistically significant difference between the ipsilateral apex–shoulder distance in postoperative imaging and the intraoperative measured apex–shoulder distance (*P* < 0.01) (Table 3). The vertical distance from the center of the contralateral and ipsilateral femoral heads to the line connecting the lower edge of the Köhler teardrop in patients with THA was 14.56 ± 2.95 mm for the contralateral group and 16.90 ± 2.99 mm for the ipsilateral group. The statistical analysis indicated a statistically significant difference (*P* = 0.005) (Supplementary Table S1), indicating the acetabular side didn't have a notable impact on bilateral lower limb length.

**Table 2:** Comparison of patients’ postoperative bilateral lower extremity length outcomes.

Group	Number of patients	Lower extremity length, mm		*P* value
		Contralateral group (mean ± SD)	Ipsilateral group (mean ± SD)	
THA	27	43.87 ± 5.59	44.64 ± 5.43	0.61
HA	25	45.18 ± 7.82	45.16 ± 6.43	0.99
Total	52	44.50 ± 6.72	44.89 ± 5.88	0.75

**Table 3:** Comparison of the ipsilateral apex–shoulder distance in postoperative imaging with the intraoperative measured apex–shoulder distance in patients (*n* = 52).

Group	Number of patients	The ipsilateral apex–shoulder distance, mm		*P* value
		Postoperative imaging measurement (mean ± SD)	Intraoperative measurement (mean ± SD)	
THA	27	18.79 ± 3.29	26.61 ± 2.45	<0.01
HA	25	20.14 ± 3.73	28.00 ± 3.11	<0.01
Total	52	19.44 ± 3.54	27.28 ± 2.85	<0.01

## Discussion

The main objectives of hip arthroplasty for femoral neck fractures in the elderly are to reduce pain, restore motor anatomy, and provide good gait and function. Bilateral leg length discrepancy is a relatively common postoperative complication that leads to postoperative patient dissatisfaction and potential medical disputes. Therefore, surgeons should pay attention to the assess of bilateral leg length discrepancy before, during, and after arthroplasty (11–13). Adequate preoperative planning is the key to successful surgery, and it includes preoperative clinical assessment, imaging measurement, and mold measurement. Preoperative mold measurements can significantly increase the success rate of hip arthroplasty by predicting the type of prosthesis, detecting possible anatomical variants, and allowing the surgeon to prepare the appropriate tools and implants in advance; it helps the surgeon to make fewer errors when there is a discrepancy between the real and mold prosthesis sizes. This helps to reduce the duration of surgery and avoid intraoperative risks (12–14). At present, preoperative planning of THA in China is still based on x-ray film mold measurement or two-dimensional (2D) preoperative planning software, which are often inaccurate owing to inadequate magnification, differences in photography projection angles, cumbersome operation, and improper prosthesis model numbers and types, resulting in a high incidence of postoperative THA complications, significantly affecting the outcome of THA surgery (15–20). CT-based preoperative planning has more stable inter- and intra-group consistency, as well as high repeatability and accuracy (21, 22). Representative software includes Mimics from Materialize (Belgium) (23), ZedHip/ZedKnee from LEXI (Japan) (21), and HipPlan from Symbios (Sweden) (24). However, all these software programs require manual segmentation of the CT images and are more complex than 2D preoperative planning software. The average duration for preoperative planning using these programs is approximately 24 min per patient (25), whereas in the present study, the AIHIP software was used to significantly reduce the preoperative planning duration, which was only approximately 5 min per patient.

The anatomical landmark positioning measurement method is simple and quick. By using the patient's inherent anatomical landmarks as a reference, the implant position is matched with the original anatomical position to restore the original functional anatomy. Kim et al. (10) used preoperative molds to determine the same center of hip rotation *via* preoperative mold measurements. Among the 156 femoral neck fracture patients, 114 underwent HA, and 42 underwent THA. The researchers used three different anatomical landmarks, i.e., the highest point of the lesser trochanter, the highest point of the greater trochanter, and the apex of the medial fracture end, to determine the osteotomy level. The reliability of the anatomical markers for the bilateral leg length discrepancy after hip arthroplasty for femoral neck fractures was compared. Wang et al. (26) determined the osteotomy line and the depth of prosthesis implantation by measuring the diameter of the contralateral femoral head and the distance from the center of the femoral head to the apex of the lesser trochanter. A satisfactory bilateral lower extremity length was obtained after HA in 47 elderly patients. We used a new “shoulder-to-shoulder” manual anatomical marker positioning method on the femoral side, which involves matching the “shoulder” of the prosthesis with the “shoulder” of the femur to restore the patient's pre-fracture anatomy, achieving the patient's inherent anatomical alignment. In this study, there was no statistically significant difference in the postoperative bilateral lower extremity length between the two surgical approaches (Table 2). In this study, the AIHIP system was used for preoperative planning, and the specific use of intraoperative prosthesis matched the preoperative AIHIP planning. The vast majority of the patients who participated in this study, the bilateral leg length discrepancy was within 5 mm, the postoperative bilateral lower extremity length satisfaction level was high, and the functional anatomy of the original joint was restored, which can be used as a reference in hip arthroplasty. However, the difference between the intraoperative and postoperative imaging measurements of the apex–shoulder distance in this study was statistically significant (Table 3) and the average difference reaches to about 8 mm, indicating that the periosteum and other soft tissues attached to the femoral trochanter have certain thicknesses and that the thicknesses of these tissues vary among people. To use the bony anatomical landmarks of the femoral greater trochanter as reference marks for the depth of femoral prosthesis penetration during surgery, the periosteum and attached soft tissues on the greater trochanter must be completely removed during surgery, which might cause further injuries to the patient and impair the early postoperative function of the hip joint. Therefore, Kim et al. (10) suggested that it is difficult to accurately determine the osteotomy level by selecting the anatomical mark of the highest point of the greater trochanter.

In this study, the “shoulder” of the femoral side was identified during surgery as the anatomical reference mark for osteotomy and femoral stem prosthesis implantation to ensure accurate osteotomy and an accurate depth of prosthesis implantation. It was combined with preoperative AIHIP planning ball-joint model number fitting for reduction to confirm the model number of the femoral head prosthesis. Wang et al. (26) suggested that accurate osteotomy and measurement of the femoral side can reduce the bilateral leg length discrepancy after THA. In this study, the femoral “shoulder” was identified as the anatomical marker (Figure 2B), and the proximal end of the template was placed at the “shoulder” of the marked femur (Figure 2D), according to the osteotomy template, the osteotomy line was marked on the femoral neck, and then the osteotomy was performed accurately. The longitudinal change of femoral head length from short head to long head or from short head to long head is only 2.45 mm (Figure 1D), which indicates the influence of femoral head prosthesis on the length of both legs after hip replacement, but the impact is limited. In the present study, there was no statistically significant difference between THA and HA with regard to the bilateral leg length discrepancy. The results indicated that the new femoral “shoulder-to-shoulder” manual anatomical landmark localization can lead to more satisfactory postoperative lengths for both lower limbs.

The present study has limitations. This was a single-center prospective study with a limited sample size, and all patients received hip arthroplasty using a posterolateral approach for femoral neck fractures. Thus, the method was not applicable for the direct anterior approach (DAA) or anterolateral approach, and it might also not be extended to patients with femoral head necrosis, hip osteoarthritis, or hip dysplasia. Besides, although we have compared the “shoulder to shoulder” anatomical marker localization method with traditional methods (contralateral contrast method and shuck test method) in a small-scale retrospective study (27), the multicenter trial with larger cohorts would need to be conducted to further validate the utility of this novel method. Finally, all prostheses in this study were provided by Johnson & Johnson (USA), and two kinds of femoral stems (Corail, DePuy, Warsaw, IN, USA; Summit, DePuy, Warsaw, IN, USA) were included, hence the proposed anatomical landmarks applicability might be limited to the stems of some specific makes.

## Conclusion

The novel method of manual positioning based on anatomical mark (shoulder-to-shoulder) for femoral neck fractures in hip arthroplasty is simple and accurate, making it effective for preventing postoperative bilateral leg length discrepancy.

## Data Availability

The raw data supporting the conclusions of this article will be made available by the authors, without undue reservation.
