# Supplementary material for: A novel method of manual positioning based on anatomical mark (shoulder-to-shoulder) to prevent postoperative leg-length discrepancy for femoral neck fractures in hip arthroplasty
Source: Front Surg. 2022 Nov 1;9:1030657. doi: 10.3389/fsurg.2022.1030657 (PMC9663648; doi:10.3389/fsurg.2022.1030657)
Supplement: Supplementary file 1 [file Table1.docx]

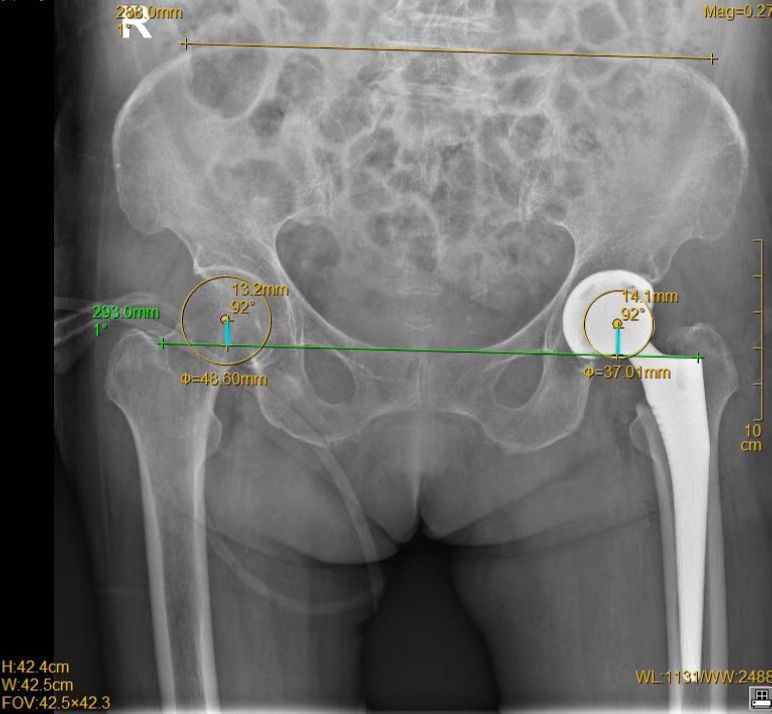


**Fig. S1**: Postoperative vertical distance from the center of the contralateral and ipsilateral femoral heads to the line connecting the lower edge of the Köhler​ teardrop (blue line).

**Table S1. Vertical distance from the center of the femoral head to the line connecting the lower edge of the Köhler​ teardrop after THA.**

| **Group** | **n** | **Vertical distance after THA, mm (mean ± SD)** |
| --- | --- | --- |
| Contralateral group | 27 | 14.56 ± 2.95 |
| Ipsilateral group | 27 | 16.90 ± 2.99 |
| *P* value |  | 0.005 |
